# Is Sonic Hedgehog Involved in Human Fracture Healing? - A Prospective Study on Local and Systemic Concentrations of SHH

**DOI:** 10.1371/journal.pone.0114668

**Published:** 2014-12-11

**Authors:** Stefan Eipeldauer, Anita Thomas, Leonard Hoechtl-Lee, Mathias Kecht, Harald Binder, Julia Koettstorfer, Markus Gregori, Kambiz Sarahrudi

**Affiliations:** 1 University Clinic for Traumatology, Medical University of Vienna, Vienna, Austria; 2 Department of Gender Medicine, Medical University of Vienna, Vienna, Austria; Van Andel Institute, United States of America

## Abstract

**Introduction:**

Sonic Hedgehog (SHH) is a new signalling pathway in bone repair. Evidence exist that SHH pathway plays a significant role in vasculogenesis and limb development during embryogenesis. Some *in vitro* and animal studies has already proven its potential for bone regeneration. However, no data on the role of SHH in the human fracture healing have been published so far.

**Methods:**

Seventy-five patients with long bone fractures were included into the study and divided in 2 groups. First group contained 69 patients with normal fracture healing. Four patients with impaired fracture healing formed the second group. 34 volunteers donated blood samples as control. Serum samples were collected over a period of 1 year following a standardized time schedule. In addition, SHH levels were measured in fracture haematoma and serum of 16 patients with bone fractures.

**Results:**

Fracture haematoma and patients serum both contained lower SHH concentrations compared to control serum. The comparison between the patients' serum SHH level and the control serum revealed lower levels for the patients at all measurement time points. Significantly lower concentrations were observed at weeks 1 and 2 after fracture. SHH levels were slightly decreased in patients with impaired fracture healing without statistical significance.

**Conclusion:**

This is the first study to report local and systemic concentration of SHH in human fracture healing and SHH serum levels in healthy adults. A significant reduction of the SHH levels during the inflammatory phase of fracture healing was found. SHH concentrations in fracture haematoma and serum were lower than the concentration in control serum for the rest of the healing period. Our findings indicate that there is no relevant involvement of SHH in human fracture healing. Fracture repair process seem to reduce the SHH level in human. Further studies are definitely needed to clarify the underlying mechanisms.

## Introduction

During the last 2 decades various studies on bone regeneration have concentrated their topics on different cytokines, angiogenic factors, proteases and growth factors such as TGF beta 1 VEGF, MCS-F and many more that were considered to be relevant for fracture healing [1]–[13]. A quite new and promising molecular signalling pathway in bone repair seems to be Sonic Hedgehog (SHH), a member of the Hedgehog family. It was first isolated in 1995 in the drosophila melanogaster. SHH signalling occurs through the interaction of the morphogen with the patched1 receptor, which then activates the Gli family of transcription factors. Evidence suggests, that the SHH pathway plays a significant role in vasculogenesis. Transgenic over expression of SHH in the dorsal neural tube of zebra fish results in hyper-vascularisation of the neuroectoderm during embryogenesis. Furthermore, zebra fish embryos lacking SHH activity show no arterial differentiation [14]. Recently, several publications indicated the importance of SHH in postnatal vascularisation processes [15], [16]. SHH plays a pivotal role in limb development during embryogenesis. In some in vitro and animal studies SHH has already proven its potential for bone regeneration [17]–[19]. Miyaji et al showed the early expression of SHH mRNA post fracture in cells at callus forming sites. In contrast Indian Hedgehog was found in the medullar cavity [18]. Moreover, there is also evidence from in vitro studies that SHH is involved in the bone regeneration process in adults and initiates osteoblastic differentiation during endochondral bone formation. [16], [20].

Due to the importance of SHH signalling in angiogenesis as well as in osteogenic differentiation we hypothesized that SHH plays a role in the bone regeneration process during human fracture healing. The aim of this study was to find out whether or not, and in what concentration SHH is present in fracture haematoma and peripheral serum to draw conclusions about its importance for fracture healing. Therefore, the local and systemic levels of SHH expression after bone fracture in patients with physiological and impaired fracture healing were analysed.

## Patients and Methods

Recruitment parameters, sample collection schedule and patient demographics have already been published in detail in earlier studies [21], [22]. In brief, serum of a consecutive series of 114 patients with meta-/diaphyseal fractures of long bone (humerus, femur, lower leg and forearm) treated surgically at our institution between April 2006 and April 2008, were collected. This was approved by the Ethic Committee of the Medical University of Vienna Nr. 1697/2012. All patients gave written informed consent to be enrolled in this project and were 18 to 90 years old. Exclusion criteria were: open fractures type III according to the Gustilo classification, previous bone operations, pre-existing bone diseases except for osteoporosis, renal/liver insufficiency, malignant tumors, long term steroid treatment, immunosuppression and long term treatment with non-steroidal anti inflammatory drugs. Due to the strict selection criteria 39 patients with incomplete data were excluded from further investigation, leaving 75 patients whose data were analysed. Patients were assigned to 2 groups according to their course of fracture healing. The first group contained 69 patients (male n = 31 female n = 38, mean age: 54.2±20.5 years) with physiological fracture healing. Six patients with impaired fracture healing formed the second group of this project. All patients with impaired fracture healing suffered from atrophic non-union and therefore underwent revision surgery. All six patients achieved bony consolidation and healed after reoperation. The diagnosis of consolidation or non-union was based on exercise-induced pain and conventional x-rays or computed tomography. Non-union was defined as the absence of complete consolidation at 6 months after surgery. Due to the small number of patients with non-union and the resulting lack of statistical power, we elected to analyze the SHH levels in 2 different periods for the same patients. We first analyzed the SHH levels of these patients from fracture to non-union. Thereafter, we analyzed the SHH levels of each patients from reoperation to bony consolidation. So the individual SHH levels of each patient were compared during the phases of impaired and during the subsequent phase of successful healing.

In addition, 34 healthy volunteers (17 males, 17 females, mean age: 35.6 years) served as controls. All patients were followed up for 12 months after the operation at our out-patient clinic. The follow up examination was based on clinical and radiological examination at 0, 1, 2, 4, 6, 8, 12, 24 and 48 weeks after trauma. Fresh fracture haematoma and peripheral venous blood were also harvested in 16 patients within 24 hours after trauma.

### Blood Samples

Peripheral venous blood was obtained from each patient at 0, 1, 2, 4, 6, 8, 12, 24 and 48 weeks after surgery, while fresh fracture haematomas were harvested from only 16 of 69 patients. A single measurement of the peripheral venous blood was performed in the controls. The samples were immediately centrifuged at 1000× g for 10 min at 4°C and the resulting serum was stored at – 80°C until analysis.

### Measurement of SHH

SHH concentrations were measured with a commercially available antibody (Biomedica Medizinprodukte Co KG, Vienna, Austria) in enzyme-linked immuno sorbent assay (ELISA). All analytical steps were performed according to the manufacturers recommended protocol. The SHH assay specifically detects the biologic active form of the protein. Concentrations are presented as mean of duplicate measurements.

### Statistical Analysis

Comparisons between independent groups of continuous variables were performed by nonparametric Mann–Whitney U-test. The nonparametric Wilcoxon-test for paired samples was used for comparisons of trial patient serums with fracture haematomas as well as the serums of patients with impaired healing before and after re-operation. Statistical analyses were performed using IBM SPSS for Windows 19.0. Data are presented as means ± SEM (standard error of the mean). The statistical significance level was set at p<0.05.

## Results

### Local Sonic Hedgehog Concentration

**Figure 1:**
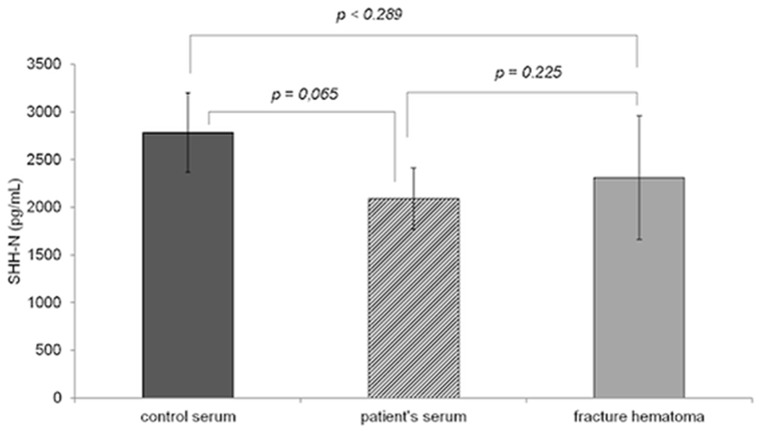
Comparison of the mean SHH concentrations between patient's serum (n = 69), control serum (n = 34) and fracture haemtoma (n = 16).

Local SHH concentration measured in fracture hematomas was higher than the systemic concentration measured at the same time in patient's serum but below the levels of control serum. The mean SHH concentration measured in the fracture hematoma was 2312,86±648,73 pg/ml and the mean SHH concentration in patients serum was 2090,38±319,50 pg/ml (p = 0.225). Mean SHH concentration in control serums was 2783,66±416,30 pg/ml. There were no significant differences between control serums and fracture haematomas (p<0.289), or patient's serum (p = 0,065). Fig. 1 demonstrates the local and serum SHH concentrations immediately after fracture.

### Systemic Sonic Hedgehog Concentrations in Patients with Physiological Fracture Healing

**Figure 2:**
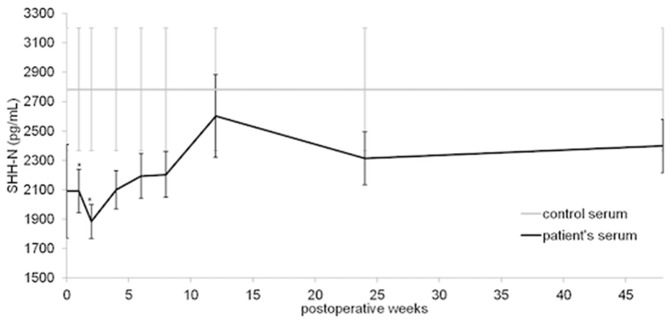
SHH serum concentrations (mean ± SEM) in controls (n = 34) and in patients with long-bone fractures and physiological healing (n = 69). Asterisks indicate significant differences in SHHconcentrations between patients and controls.

Mean serum SHH concentrations were 2090,38±319,50 pg/ml immediately after fracture. Thereafter, the time course of SHH serum concentration was characterized by a continuous increase of the SHH levels until the 12th week. The mean SHH serum level was 2091,73±147,13 pg/ml at 1 week after fracture. Week 2 was characterized by a decrease of the SHH level (1884,86 pg/ml). Serum levels then again increased to peak at 12 weeks after trauma (2601,09±280,99 pg/ml). The concentrations decreased thereafter and reached 2399±181,54 pg/ml at 48 weeks after fracture. The comparison between the patients' SHH level and the control serum revealed lower levels for the patients at all measurement time points (Fig. 2). Significantly lower concentrations were observed at weeks 1 and 2 after fracture (week 1 p = 0.028, week 2 p = 0.016).

### Systemic Sonic Hedgehog Concentrations in Patients with Impaired Fracture Healing

Apart from the overall lower concentration in patients with impaired healing, the time course of SHH level in these was similar to the patients with physiological healing. The lowest concentration was observed at week two after fracture (1884.86±115.65 pg/ml) and the peak concentration was reached at week 12 (2601.09±281 pg/ml), nearly identical to the SHH course in patients with physiological healing. Due to the small sample size of the group with impaired fracture healing, the comparison between the groups was only possible for weeks 1, 2, 4, and 12 after operation for trauma. During theses time points no statistically significant differences between patients with physiological and impaired healing were observed. Likewise, the comparison between the SHH level of the patients with impaired fracture healing and the control serum revealed no significant difference.

### Systemic Sonic Hedgehog Concentrations in Patients with Impaired Fracture Healing after Reoperation for Non- Union

**Figure 3:**
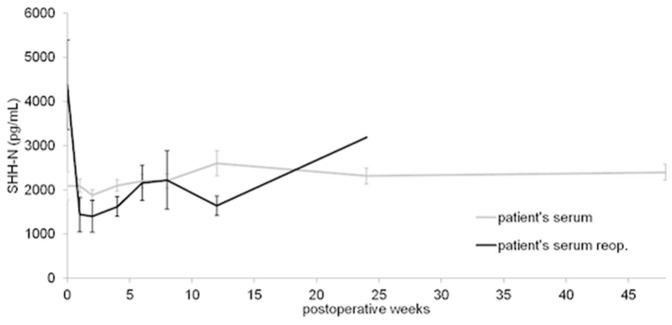
SHH serum concentrations (mean ± SEM) of patients with long-bone fractures and impaired bone healing after initial fixation for fracture (non-union) (n = 4) and the same patients after re-operation for non-union (non-union after reop.) (n = 4).

Only 4 patients had a complete data set and were subsequently included in this analysis. All 4 patients underwent reoperation for non-union, and consequently achieved proper bony consolidation and healed after the revision surgery. After surgical intervention for non-union, serum concentrations decreased rapidly at 1 week to reach its minimum at week 2 (1404±363 pg/ml). This was then followed by a constant, clear increase until week 8 after reoperation (2225,33±154,09 pg/ml). The concentrations decreased again at week 12 (1642.33±221.9 pg/l). Fig. 3 demonstrates the mean SHH concentrations of the patients with impaired healing before and after re-operation for non-union.

## Discussion

To the best of our knowledge, this is the first report on the local and systemic presence of SHH during fracture healing in humans. Moreover, this is the first study ever to report SHH serum concentrations in adults. A review of the literature shows that nearly the entire knowledge of SHH is derived from in vitro or animal studies. Only very little data on SHH in adults exist, hence the role of this protein in adult humans is completely unknown. Based on the reported experimental data on the important role of SHH on the limb development during embryogenesis and its role in the bone regeneration and angiogenesis, we hypothesized that SHH plays a role in the bone regeneration process during the human fracture healing. We further hypothesized that serum SHH concentrations differ in patients with physiological and impaired fracture healing.

Serum SHH levels in healthy adult controls were initially analysed to obtain reference values. To our knowledge, based on literature search, there are no reported data on the serum concentration of SHH in adults. Serum SHH levels were recently reported in in 44 children with autism and 40 healthy children of 3 to 9 years of age [23]. The authors found, that serum SHH levels in children with mild and severe forms of autism were significantly higher than the SHH level in healthy children. The reported serum level in healthy children was 2.6±2 pg/ml. Astonishingly, and against our expectation serum SHH concentrations measured in our healthy adult controls were 1000 fold higher (2783.66±416 pg/ml).

The next issue addressed in this study was the possible involvement of SHH in human fracture healing. We analysed the SHH concentration in fresh fracture haematomas obtained immediately after fracture and compared it to the SHH concentrations in the serum of the same patients obtained at the same time to investigate if SHH is initially released from within the fracture site or is brought there from peripheral blood. SHH was found to be present in both, the fracture haematoma and the patients' serum immediately after fracture.

We found slightly higher concentrations in fracture haematomas than in patients' serums, but without statistical significance. We therefore found no indication that SHH is released from within the fracture and not brought into the fracture by the peripheral serum. Moreover, local and systemic concentrations measured in patients were lower than the concentrations measured in the control serum. Likewise, the SHH serum concentrations measured during fracture healing were also lower than the controls. Interestingly, concentrations during the inflammatory phase were significantly lower than in the controls'. These data are inconsistent with previously reported experimental data and suggest, that the repair processes during fracture healing seem to reduce the release of SHH. Considering these findings the question of the underlying mechanism arises. It is known that SHH is involved in early chondrogenesis [24]-[27] and in early osteoblastic differentiation [28]. SHH gene has been reported to be closely localized with bone morphogenetic protein (BMP) in the early stage limb bud [29]. SHH has also been reported to be an important signalling factor for limb formation during embryogenesis and some studies report an effect of SHH on bone regeneration after fracture by controlling osteoclast formation and osteoblast proliferation [30], [31]. SHH and BMP modulate each others' gene expression [26], [27], [32] and their action through signalling pathways via Gli [33], a mediator of hedgehog protein. In vitro, SHH causes proliferation and differentiation of mesenchymal stem cells into the osteoblastic lineage by up-regulating BMPs via SMAD signalling [30]. In a previous study, the expression of mRNAs for SHH at the fracture site in mice tibiae was investigated [18]. Transcripts for SHH were not detected in bone pre fracture but they were identified in proliferating callus-forming cells in the periosteum and the surrounding tissue, and in the medullary cavity prior to apparent new cartilage and bone formation. Gli 1 and SHH transcripts were found on day 2. On day 12, SHH was no longer detected. These studies provide strong evidence that SHH modulates and affects bone regeneration in animal models. Thus it is difficult to understand why SHH concentrations are decreased during fracture healing in humans.

In another recent study the effect of SHH to improve bone defect healing was tested [34]. SHH, BMP-2 or a combination of both added to β-tricalcium phosphate (β-TCP) was used to treat a femoral defect in rats. The authors observed that the addition of SHH delayed bone healing and resulted in a lower stiffness even in combination with BMP-2. Hypothetically, the results of Warzecha et al. could be interpreted that the presence of SHH in the fracture site could lead to healing insufficiency. The results of this German group might help to understand the significant reduction of SHH level observed in our patients. Nevertheless, we must once again emphasize the hypothetical character of this statement.

## Conclusions

In summary, this is the first report on the local and systemic concentration of SHH in human fracture healing. Moreover, this is the first study to describe SHH serum levels in healthy adults. We are aware of the fact that this study is purely descriptive. Another main limitation of this study remains the small size of the patients with impaired fracture healing. However, the strengths of our study include the large number of patients with physiological healing, as well as its longitudinal design. We found significantly reduced levels of SHH during the inflammatory phase of fracture healing. SHH concentrations in fracture haematoma and serums were lower than in control serums for the entire healing period, though not statistically significant. Our findings indicate that there is no relevant involvement of SHH in human fracture healing, but further studies are definitely needed to clarify the underlying mechanisms.
